# GC/MS analysis and potential synergistic effect of mandarin and marjoram oils on *Helicobacter pylori*

**DOI:** 10.1080/14756366.2022.2081846

**Published:** 2022-05-31

**Authors:** Rawah H. Elkousy, Nada M. Mostafa, Ahmed M. Abd-alkhalek, Mahmoud A. El Hassab, Sara T. Al-Rashood, Wagdy M. Eldehna, Omayma A. Eldahshan

**Affiliations:** a Department of Pharmacognosy, Faculty of Pharmacy (for Girls), Al-Azhar University, Cairo, Egypt; b Department of Pharmacognosy, Faculty of Pharmacy, Ain Shams University, Cairo, Egypt; c Faculty of Medicine (for Boys), Al Azhar University, Cairo, Egypt; d Department of Medicinal Chemistry, Faculty of Pharmacy, King Salman International University (KSIU), Ras Sedr, Egypt; e Department of Pharmaceutical Chemistry, College of Pharmacy, King Saud University, Riyadh, Saudi Arabia; f School of Biotechnology, Badr University in Cairo, Badr City, Egypt; g Department of Pharmaceutical Chemistry, Faculty of Pharmacy, Kafrelsheikh University, Kafrelsheikh, Egypt; h Center for Drug Discovery Research and Development, Faculty of Pharmacy, Ain Shams University, Cairo, Egypt

**Keywords:** Marjoram oil, petitgrain mandarin oil, *Helicobacter pylori*, essential oils, clarithromycin

## Abstract

*Helicobacter pylori* can cause chronic gastritis, peptic ulcer, and gastric carcinoma. This study compares chemical composition and *anti-H. pylori* activity of mandarin leaves and marjoram herb essential oils, and their combined oil. GC/MS analysis of mandarin oil revealed six compounds (100% identified), mainly methyl-*N*-methyl anthranilate (89.93%), and 13 compounds (93.52% identified) of marjoram oil, mainly *trans*-sabinene hydrate (36.11%), terpinen-4-ol (17.97%), linalyl acetate (9.18%), and caryophyllene oxide (8.25%)). Marjoram oil (MIC = 11.40 µg/mL) demonstrated higher activity than mandarin oil (MIC = 31.25 µg/mL). The combined oil showed a synergistic effect at MIC of 1.95 µg/mL (same as clarithromycin). *In-silico* molecular docking on *H. pylori* urease, CagA, pharmacokinetic and toxicity studies were performed on major compounds from both oils. The best scores were for caryophyllene oxide then linalyl acetate and methyl-*N*-methyl anthranilate. Compounds revealed high safety and desirable properties. The combined oil can be an excellent candidate to manage *H. pylori.*

## Graphical Abstract

**Figure:**
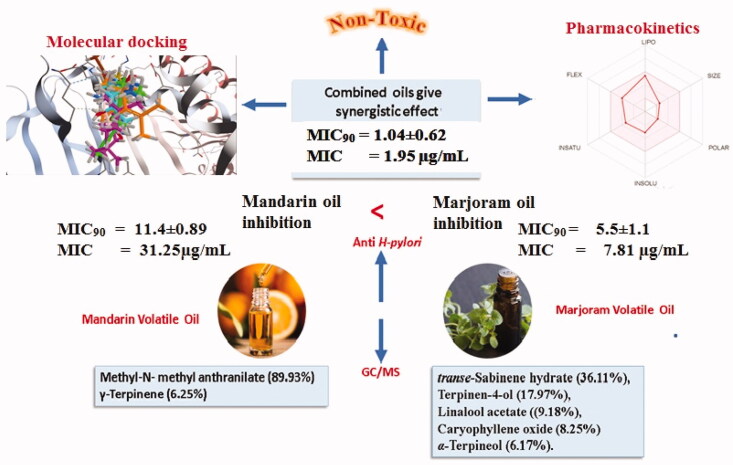

## 1. Introduction

Despite enormous progress in medicinal strategies for the treatment of many human health problems, infectious diseases continue to pose a significant threat to public health^1^. *Helicobacter pylori* is an extracellular gram-negative spiral bacterium, that is now recognised as a major cause of gastroduodenal diseases such as chronic gastritis, which affects nearly everyone and leads to peptic ulcers or gastric adenocarcinoma, the second most common cause of cancer death worldwide^2^^,^^3^. Standard medications can cure the infection in more than 80% of *H. pylori*-infected patients. Patient compliance, antibiotic resistance, and recurring infections, on the other hand, are all the major concerns that limit the use of antibiotics in the treatment of *H. pylori* infection that needs to be addressed^4^^,^^5^.

Natural products are reported to demonstrate various biological activities^6–10^ and as promising antimicrobials^11^^,^^12^. The importance of plant-based products for disease treatment is growing exponentially due to the increased incidence of adverse drug reactions^13^ and the development of microbial resistance to the available antimicrobial drugs^14^.

Essential oils derived from aromatic and medicinal plants have recently gained popularity and great scientific interest as they are a part of traditional medicine predominating all over the globe for the alleviation of various health problems. Essential oils have been shown to possess potential antibacterial, antifungal, antiviral, anticancer, and antioxidant properties such as cinnamon, orange, lemon, pepper, thyme, and *Schinus*^15–20^. Besides, they act as an important milestone in alternative medicine as well as natural therapies^1^. Therefore, it is reasonable to expect that a variety of plant compounds in these oils have antimicrobial effects.

Among several essential oils that may be useful as antimicrobial agents, marjoram oil (*Origanum majorana* L., Lamiaceae) is an aromatic medicinal plant with the greatest potential for industrial applications because it shows different biological activities, including antibacterial, antifungal, antihypertensive, anti-inflammatory, and antioxidant properties^21–23^. *Origanum majorana* leaves and essential oil have been claimed to be useful for the treatment of respiratory and gastrointestinal problems^24^. It is one of the most popular spices used in cooking, arousing interest not only in the use of its leaves but also in its essential oil for therapeutic purposes^25^.

On the other hand, the genus *Citrus* (Rutaceae) has been one of the most popular and commercially important crops for thousands of years. Citrus fruits are known for their nutritional values as an excellent source of vitamin C, their unique flavour, and their medicinal properties^26^. Interestingly, essential oil (EO) is the most vital by-product of citrus processing. Petitgrain mandarin essential oil is extracted from *Citrus reticulata* leaves. It could relieve stress and digestive problems while helping with flatulence, diarrhoea, and constipation. It is mostly used to increase circulation to the skin, reducing fluid retention and helping prevent stretch marks. Mandarin oil is used to calm the nervous system and has a tonic effect^27^^,^^28^. Moreover, it showed broad-spectrum antibacterial and antifungal agents. It inhibited the growth of several bacterial and fungal strains^29–31^. Furthermore, petitgrain mandarin essential oil showed potential antioxidant, anticancer (HL-60 and NB4), and radical scavenging activities^32^.

The increasing emergence of *H. pylori* infections worldwide as well as the emerging tolerance against most currently available antibiotics has necessitated the urgent need to discover novel and highly effective antimicrobial regimens due to the lack of therapies available to control *H. pylori* infections. Meanwhile, it has been noticed that a few plants have been investigated recently for their *H. pylori* bactericidal activity. Antibacterial drug interactions can change the efficacy and either synergistic or antagonistic action, interaction between different compounds can lead to the reduction of the inhibitory activity^33^.

This has driven our interest to assess the constituents of essential oils of marjoram (*Origanum majorana* L.) and mandarin leaves by using GC-MS, as well as evaluate the synergistic *anti-H. pylori* activity *in-vitro* of these oils, as compared to clarithromycin. An *in-silico* study was performed, where molecular docking was carried out on the major compounds identified from both oils on *H. pylori* virulent factors domains such as urease and CagA. Further *in-silico* pharmacokinetic and toxicity studies were performed on these major components to determine their safety margins and properties.

## 2. Materials and methods

### 2.1. Essential oils

The whole herb of *Origanum majorana* was subjected to steam distillation for 5 h. The oil produced has a pale yellow colour and herbaceous sweet odour. *Citrus reticulata* oil was prepared by water distillation of the leaves using the Clevenger apparatus for 5 h. Its colour is pale yellow and of intensely sweet and fresh scent. Both oils were purchased from Somitt Aromatic Company that were kept in dark bottles.

### 2.2. Analysis of essential oils by gas chromatography

#### 2.2.1. GC/FID analysis

The GC/FID analyses were carried out on a Varian 3400 apparatus (Varian GmbH, Darmstadt, Germany) equipped with an FID detector and an Rtx-5MS fused-bonded silica column (30 m x 0.25 mm i.d., film thickness 0.25 µm; Ohio Valley, Ohio, USA); the operating conditions were: The initial column temperature was kept at 45 °C for 2 min (isothermal), and then programmed rising at a rate of 5 °C/min to 300 °C and held for 5 min. Detector and injector temperatures were 300 °C and 250 °C, respectively. The sample volume was 0.03 µl, Helium carrier gas flow rate was 2 ml/min. Peak Simple 2000 chromatography data system (SRI Instruments, Torrance, USA) was used for recording and integrating the chromatograms.

#### 2.2.2. GC/MS analysis

The analyses were carried out on a Hewlett Packard gas chromatograph (GC HP 5890 II; Hewlett Packard GmbH, Bad Homburg, Germany) equipped with the same column and conditions as for the GC/FID. The capillary column was directly coupled to a quadrupole mass spectrometer (SSQ 7000; Thermo-Finnigan, Bremen, Germany). The injector temperature was 250 °C. Helium carrier gas flow rate was 2 ml/min. All the mass spectra were recorded with the following analytical conditions: filament emission current, 60 mA; electron energy, 70 eV; ion source temperature, 200 °C; and scan range was from 40 to 400 Amu. The diluted samples (0.5% v/v n-hexane used as solvent) were injected with split mode (split ratio, 1:15). Compounds were identified by comparison of their mass spectral data and retention indices with Wiley Registry of Mass Spectral Data 8th edition and NIST Mass Spectral Library (December 2005). The identification was further confirmed by the calculation of the retention indices (RI) relative to a homologous series of *n*-alkanes (C6 - C22), under identical experimental conditions, as well as matching with the literature^34–37^.

### 2.3. Assessment of the *anti-Helicobacter pylori* activity

#### 2.3.1. Determination of the minimal inhibitory concentration (MIC)

The micro-well dilution method was used to evaluate the antibacterial activity of the Marjoram and Mandarin oils against *Helicobacter pylori* (ATCC 43504, the reference strain being obtained from the American Type Culture Collection) adopting the NCCLS guidelines (1998) and as previously described by Cerda et al.^38^. 100 µg of the tested samples were combined with 100 µL of 20% (v/v) bacterial suspensions (OD at 600 nm = 1.0) in a flat bottomed 96-well microplate. Serial two-fold dilutions of the oils and the standard were prepared directly in a sterile 96-well microtiter plate. Deionised water was used as a negative control meanwhile clarithromycin was used as a positive control. The reaction mixture was incubated using Mueller-Hinton broth. After incubation at 37 °C for 3 days under microaerophilic conditions (10% CO_2_ and 80% humidity). 25 µL of 10 mM 3–(4,5-dimethyl-thiazol-2-yl)-2,5-diphenyl-tetrazolium bromide (MTT) freshly prepared in water were added into the mixture (final volume was 225 µL) to each well and incubate for 30 min. The developed purple colour was measured at 550 nm using a microplate reader^1^. All tests were performed in triplicate. Inhibition (%) was calculated as follows: [(Initial control absorbance-final absorbance)/(Initial control absorbance)] × 100.

The Agar dilution checkerboard method is used to evaluate the synergic action of both essential oils. The MIC90, the concentration of samples with 90% inhibition was calculated from dose-response curves. The MIC values were assessed in triplicate, using an automatic ELISA microplate reader.

### 2.4. *In-silico* studies

#### 2.4.1. Molecular docking

The X-ray 3D structures of human *H. pylori* Urease and Cag A oncogenic proteins were downloaded from the protein data bank using the following IDs: 1e9y and 4dvy, respectively. All the docking studies were conducted using MOE 2019^39^ which was also used to generate the 2D and 3D interaction diagrams between the docked ligands and their potential targets. In the beginning, the two enzymes and the seven isolated major compounds were prepared using the default parameters. The active site of each target was determined and the seven isolated compounds were saved into a single file with MDB extension. Finally, the docking was finalised by docking the mdb file containing the seven compounds into the active site of both the enzymes.

#### 2.4.2. *In-silico* toxicity and ADME prediction

Both the Toxicity and pharmacokinetic properties of the seven compounds were predicted by the online servers ProTox II (https://tox-new.charite.de/protox_II/index.php?site=compound_input) and Swiss adme (http://www.swissadme.ch/index.php), respectively.

## 3. Results

### 3.1. Determination of the volatile oil compositions using GC/MS

**Figure 1.**
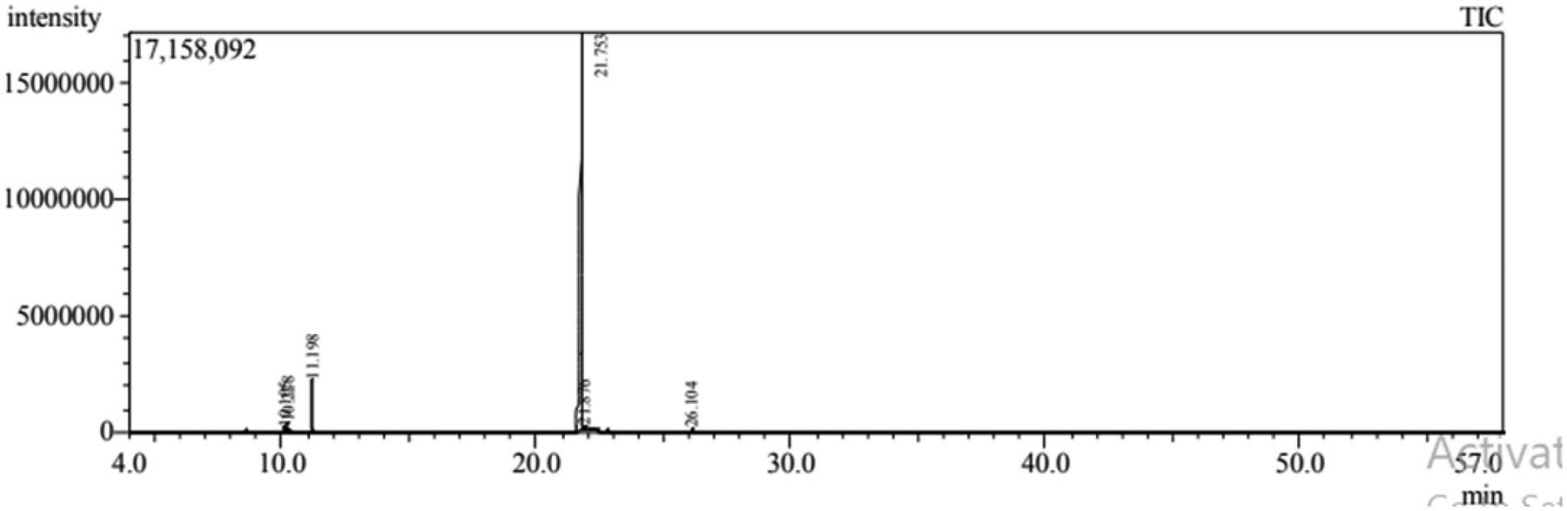
GC-MS Chromatogram of mandarin oil.

**Figure 2.**
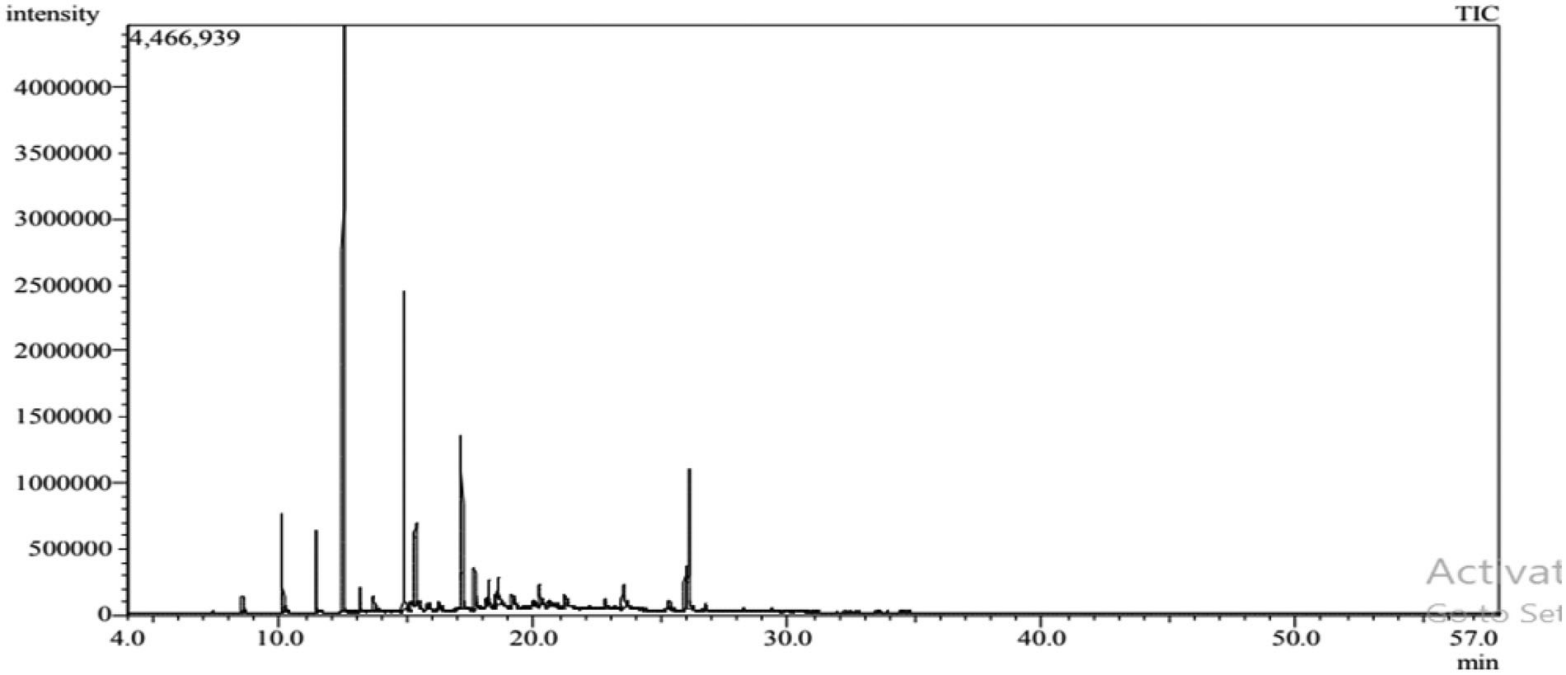
GC-MS Chromatogram of marjoram oil.

Six compounds were identified from the oil of mandarin leaves (Figure 1, Table 1). The predominant constituents of the oil were methyl-*N*-methyl anthranilate (89.93%) and *γ*-terpinene (6.25%), respectively. While 93.52% of marjoram oil was identified (Figure 2, Table 2), the major peaks were for *trans*-sabinene hydrate (36.11%), terpinen-4-ol (17.97%), linalyl acetate (9.18%), caryophyllene oxide (8.25%), and α-terpineol (6.17%), respectively.

**Table 1.** The essential oil composition of mandarin leaves oil.

Peak no.	Components	Molecular formula	Retention time (min)	RI_cal_	RI_Lit_	% Composition	Method of identification
1.	*p*-Cymene	C_10_H_14_	10.105	1015	1018	0.72	RI, MS
2.	Limonene	C_10_H_16_	10.228	1019	1022	1.35	RI, MS
**3.**	**γ-Terpinene**	**C_10_H_16_**	**11.198**	**1050**	**1055**	**6.25**	**RI, MS**
**4.**	**Methyl-*N*-methyl anthranilate**	**C_9_H_11_NO_2_**	**21.753**	**1406**	**1402**	**89.93**	**RI, MS**
5.	*β*-Caryophyllene	C_15_H_24_	21.876	1411	1418	1.11	RI, MS
6.	Caryophyllene oxide	C_15_H_24_O	26.104	1576	1572	0.63	RI, MS
	**Total identified**					**100**	

Major compounds are in bold.

**Table 2.** The essential oil composition of marjoram oil.

Peak no.	Components	Molecular formula	Retention time (min)	RI_cal_	RI_Lit_	% Composition	Method of identification
1.	Camphene	C_10_H_16_	8.530	959	953	0.65	RI, MS
2.	*p*-Cymene	C_10_H_14_	10.114	1015	1018	3.18	RI, MS
3.	*γ*-Terpinene	C_10_H_16_	11.452	1058	1059	3.51	RI, MS
**4.**	***trans*-Sabinene hydrate**	**C_10_H_18_O**	**12.506**	**1092**	**1098**	**36.11**	**RI, MS**
5.	Dehydro Sabinene ketone	C_9_H_12_O	13.137	1112	1117	1.12	RI, MS
6.	3-Isothujenol	C_10_H_18_O	13.703	1130	1134	0.82	RI, MS
**7.**	**Terpinen-4-ol**	**C_10_H_18_O**	**14.914**	**1169**	**1174**	**17.97**	**RI, MS**
**8.**	***α*-Terpineol**	**C_10_H_18_O**	**15.335**	**1183**	**1186**	**6.17**	**RI, MS**
**9.**	**Linalyl acetate**	**C_12_H_20_O_2_**	**17.163**	**1245**	**1254**	**9.18**	**RI, MS**
10.	U.I.	–	17.659	1263	–	2.55	–
11.	Bornyl acetate	C_15_H_18_O_2_	18.199	1281	1280	1.24	RI, MS
12.	3-Thujanyl acetate	C_12_H_20_O_2_	18.485	1291	1295	0.53	RI, MS
13	U.I.	–	18.560	1294	–	2.10	–
14	U.I.	–	20.200	1351	–	1.94	–
15	U.I.	–	23.502	1475	–	2.45	–
16	Spathulenol	C_15_H_24_O	25.961	1571	1577	2.24	RI, MS
**17**	**Caryophyllene oxide**	**C_15_H_24_O**	**26.103**	**1576**	**1581**	**8.25**	**RI, MS**
	**Total identified**					93.52	

U.I.: Unidentified, major compounds are in bold.

### 3.2. Determination of *anti-Helicobacter pylori* activity

**Figure 3.**
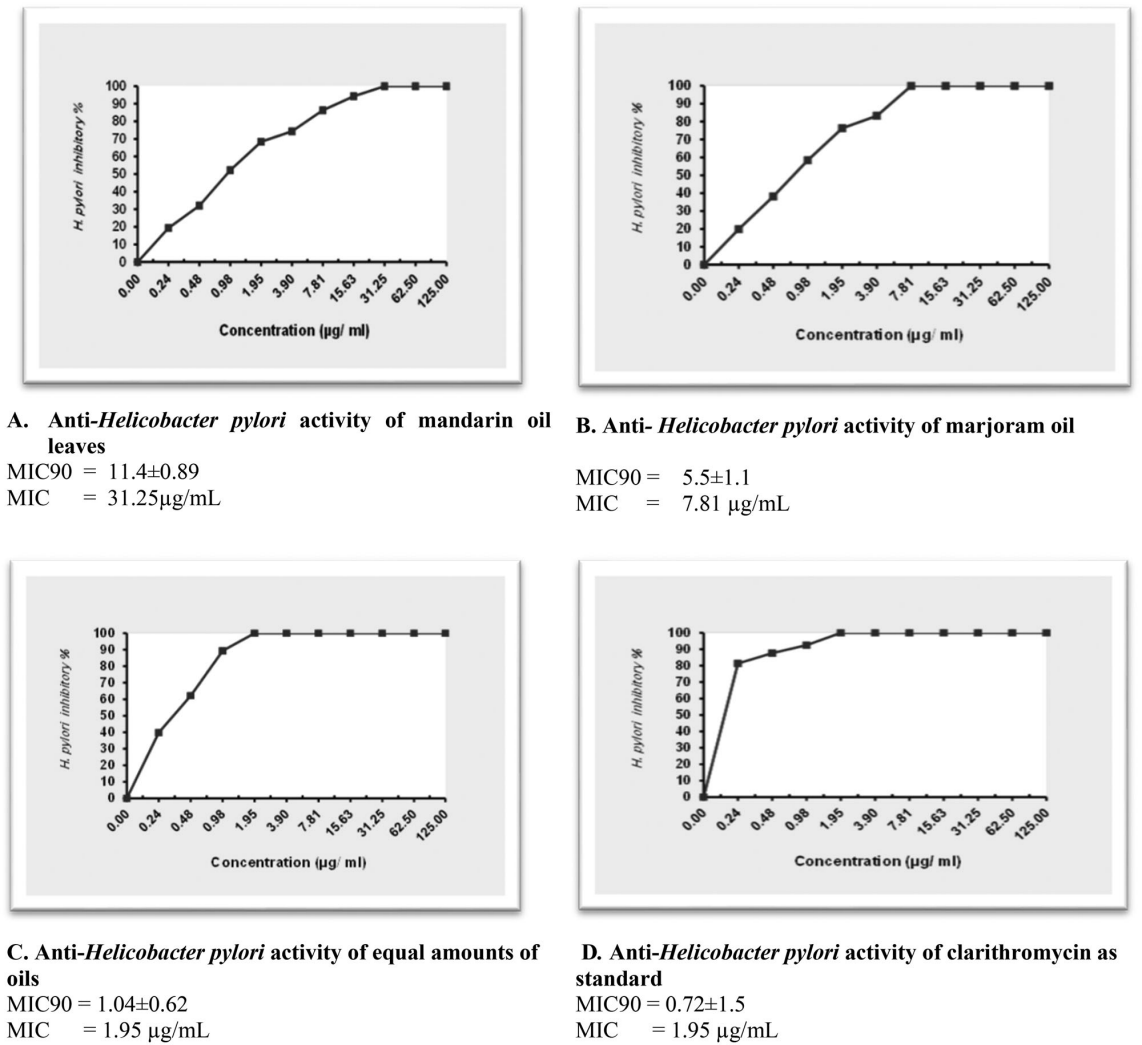
The MIC_90_ & MIC graphs of *Anti-Helicobacter pylori* activity of mandarin oil leaves (A), marjoram oil (B), equal amounts of both oils(C), and Clarithromycin (D) as standard. All determinations were carried out in a triplicate manner and values are expressed as the mean ± SD.

This study’s focal objective is to compare the antimicrobial activities of essential oils of mandarin leaves and marjoram against *Helicobacter pylori.* The MIC results for the two tested oils separately and combined with clarithromycin as a positive control are shown in Figure 3(A–D), respectively.

The results of this study revealed that marjoram oil showed higher antibacterial activity against *H. pylori* at a MIC of 11.4 µg/mL, (Figure 3B) relative to mandarin essential oil, which exhibited a MIC value of 31.25 µg/mL (Figure 3A). This may be attributed to the presence of high content of oxygenated compounds in marjoram oil that were identified as shown in (Table 2), including *trans-*sabinene hydrate (36.11%), terpinen-4-ol (17.97%), linalyl acetate (9.18%), caryophyllene oxide (8.25%), and α-terpineol (6.17%).

While methyl-*N*-methyl anthranilate (89.93%) has been identified as the major constituent of mandarin leaf oil, followed by γ-terpinene (6.25%) as shown in (Table 1). A combined mixture of both oils exhibited a potentially synergistic inhibitory effect against *H. pylori* at a MIC of 1.95 µg/ml, yielding higher inhibition (Figure 3C) relative to marjoram and mandarin oils separately. Furthermore, clarithromycin demonstrated the same MIC value (1.95 µg/mL).

### 3.3. Docking study

**Figure 4.**
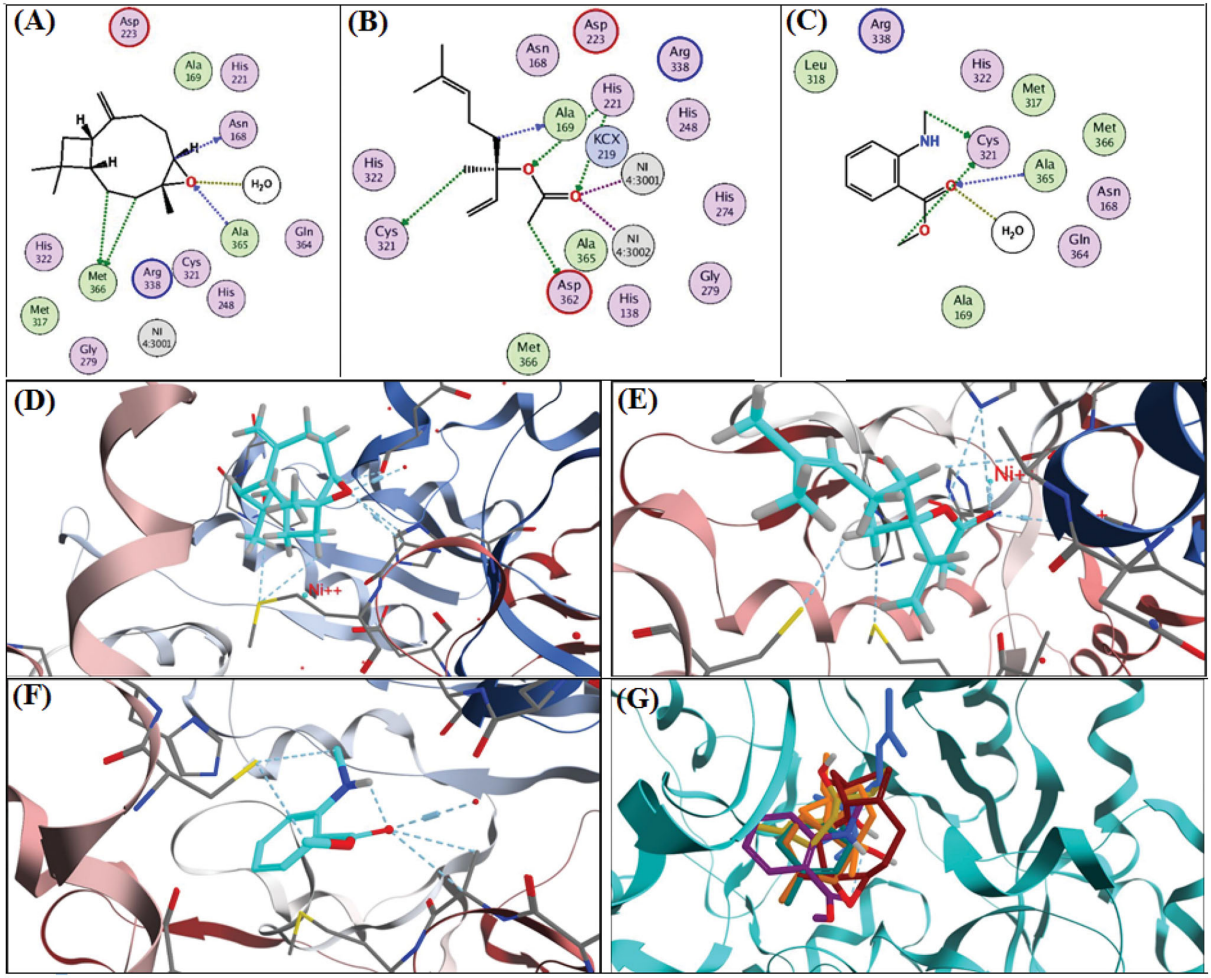
2D and 3D-binding affinities of caryophyllene oxide (A,D), linalyl acetate (B,E), methyl-*N*-methyl anthranilate (C,F) and concomitant interactions of seven major compounds identified in marjoram and mandarin oils with the active sites of H. *pylori* urease domain.

**Figure 5.**
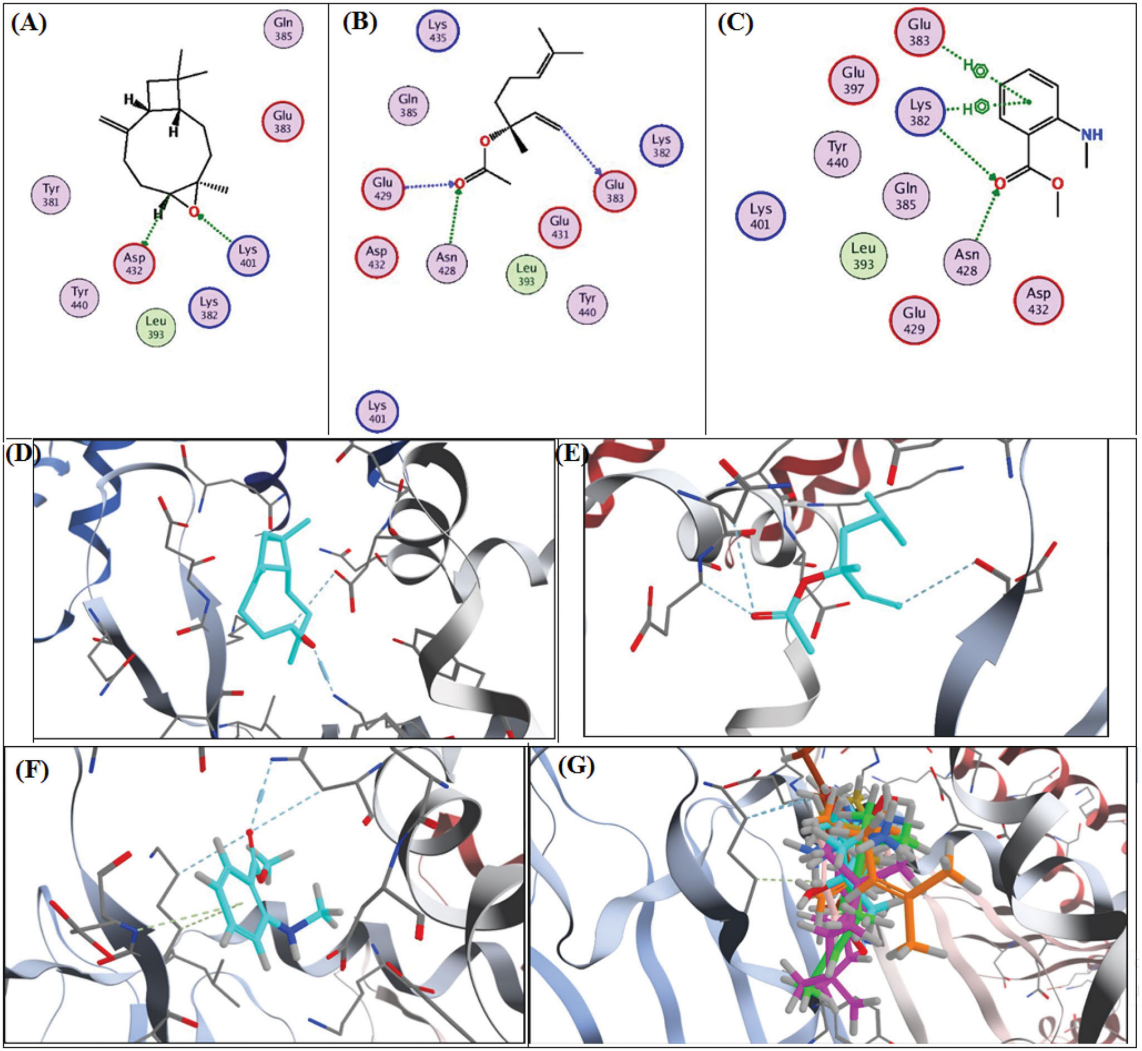
2D and 3D-binding affinities of caryophyllene oxide (A,D), linalyl acetate (B,E), methyl-*N*-methyl anthranilate (C,F) and concomitant interactions of seven major compounds identified in marjoram and mandarin oils with the active sites of H. *pylori* CagA domain.

A molecular docking study was carried out on the major compounds identified from both oils on *H. pylori* virulent factors domains such as urease and CagA. Results showed that caryophyllene oxide showed the best fitting scores followed by linalyl acetate and methyl-*N*-methyl anthranilate, as demonstrated in Table 3. The binding affinities of caryophyllene oxide, linalyl acetate and methyl-*N*-methyl anthranilate to urease and CagA are further demonstrated in Figures 4, 5, respectively. Caryophyllene oxide showed the best binding affinities to the urease enzyme by 4 hydrogen bonds (H-bonds) and solvent interaction, while it revealed an interaction with only 2 H-bonds with cagA. Linalyl acetate exhibited 5 H-bonds and 2 metal (Nickel) co-ordinates with urease and 3 H-bonds with cagA. Regarding, methyl-*N*-methyl anthranilate, it showed 3 H-bonds and solvent interaction with urease, while it demonstrated 2 H-bonds and 2 hydrophobic interactions with the active sites of CagA. Figures 4G, 5G revealed concomitant interactions of seven major compounds identified in both oils with the active sites of urease and cagA, respectively, to reveal more of the synergistic effect of these components as anti-*H. pylori.*

**Table 3.** Docking scores of marjoram and mandarin major oil compounds on *H. pylori* virulent factors domains (urease and CagA).

Compound name	Urease (1e9y)	Cag A (4dvy)
Caryophyllene oxide	−11.4	−9.5
Linalyl acetate	−9.1	−8.3
Methyl-*N*-methyl anthranilate	−8.9	−8.2
α- Terpineol	−7.6	−7.4
*trans*-Sabinene hydrate	−7.4	−6.9
Terpine-4-ol	−7.2	−7.1
γ-Terpinene	−6.6	−5.4

### 3.4. *In-silico* toxicity study

As demonstrated in Table 4 all the compounds have high margins of safety and they were predicted to have no potential toxicity.

**Table 4.** The predicted toxicity of marjoram and mandarin major oil compounds.

Compound name	Any potential toxicity	Predicted LD50
*trans*-Sabinene hydrate	2000 mg/kg	None
Terpine-4-ol	1016 mg/kg	None
Linalyl acetate	12000 mg/kg	None
Caryophyllene oxide	5000 mg/kg	None
α-Terpineol	2830 mg/kg	None
Methyl-*N*-methyl anthranilate	2910 mg/kg	None
γ-Terpinene	2500 mg/kg	None

### 3.5. Pharmacokinetics study

It is important for therapeutic candidates to have both acceptable pharmacokinetics and pharmacodynamics profiles. Accordingly, the pharmacokinetic profiles of the seven major compounds were computed using the online server of swiss adme. As depicted in Table 2. All the compounds were predicted to have high GIT absorption making them excellent oral candidates against *H. pylori*.

**Figure 6.**
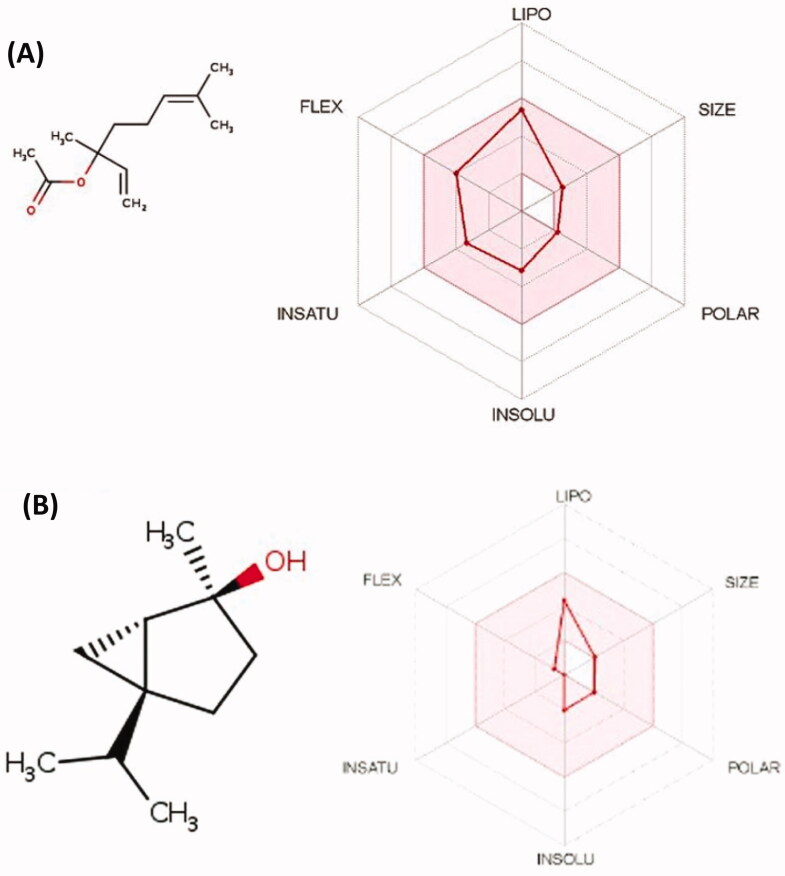
The predicted physicochemical properties for selected compounds, such as linalyl acetate (A) and *trans*-sabinene hydrate (B).

This high bioavailability of the seven compounds is attributed to their desired physicochemical properties including FLEX (Flexibility), LIPO (Lipophilicity), INSATU (Saturation), INSOLU (Solubility), SIZE and POLAR (Polarity) as demonstrated in Table 5 and Figure 6. In addition, all compounds showed no or minimal interaction with microsomal cytochromes and then could be taken concurrently with other medications. Most importantly, no compound was found to be a substrate for the p-glycoprotein known to be one of the resistance mechanisms of *H. pylori* for existing antibiotics^40^. A worthy note, the seven compounds were aligned with all of Lipinski's rules, besides none of them had any reported Pan Assay Interference (PAINS).

**Table 5.** The predicted pharmacokinetics of marjoram and mandarin major oil compounds.

Parameter	*trans*-Sapinene hydrate	Terpine-4-ol	Linalyl acetate	Caryophyllene oxide	α-Terpineol	Methyl-*N* methyl anthranilate	γ-Terpinene
GIA	High	High	High	High	High	High	High
BBB	Yes	Yes	Yes	Yes	Yes	Yes	Yes
P-gP substrate	No	No	No	No	No	No	No
CYP1A2 inhibitor	No	No	No	No	No	Yes	No
CYP2C19 inhibitor	No	No	No	Yes	No	No	No
CYP2C9 inhibitor	No	No	No	Yes	No	No	No
CYP2D6 inhibitor	No	No	No	No	No	No	No
CYP3A4 inhibitor	No	No	No	No	No	No	No
Lipinski Violations	No	No	No	No	No	No	No
PAINS	No	No	No	No	No	No	No

GIA (gastrointestinal absorption), BBB (Blood Brain Barrier), PgP (P-glyco pro- tein transporter), CYP1A2, CYP2C19, CYP2C9, CYP2D6 and CYP3A4 are the five forms of cytochromes P450 (CYP). PAINS (Pan Assay Interference).

## 4. Discussion

The continuous evolution of many drawbacks with the current therapies for *H. pylori,* such as the prevalence of antibiotic-resistant, drug interventions, side effects, and poor satisfaction, all highlight the search for safe and effective non-antibiotic alternative medicines^5^. This has driven our interest in evaluating the bactericidal activity of marjoram and mandarin oils against *H. pylori.* At present, interest in essential oils has increased because of their bactericidal activity against several bacteria without the marked toxic effects of synthetic drugs.

Bactericidal activity is a well-known property of volatile oils, particularly those of marjoram and mandarin. Numerous studies have confirmed the antimicrobial activity of marjoram essential oils^21^^,^^22^^,^^25^^,^^41^^,^^42^ and mandarin leaf essential oils^26^^,^^43–46^. This study investigates the bactericidal activity of the hydro-distilled essential oils of the leaves of mandarin and marjoram. The results of this study revealed that marjoram oil showed a higher effect against *H. pylori* than mandarin essential oil. This may be attributed to the presence of high content of oxygenated compounds identified in marjoram oil, including *trans-*sabinene hydrate (36.11%), terpinen-4-ol (17.97%), linalyl acetate (9.18%), caryophyllene oxide (8.25%), and α-terpineol (6.17%). While methyl-*N*-methyl anthranilate (89.93%) was identified as the major constituent of mandarin leaf oil, followed by γ-terpinene (6.25%).

Many studies of *in vitro* antimicrobial activity of marjoram and mandarin oils in the literature may be probably due to the action of the major compounds which have been previously tested for their bactericidal activity, such as terpinen-4-ol, α-terpineol, and γ-terpinene were found as the predominant components of the essential oils obtained from the aerial parts of *Origanum scabrum* and *Origanum microphyllum*, both endemic species in Greece, exhibited a very interesting antimicrobial profile after they were tested against six Gram-negative and Gram-positive bacteria and three pathogenic fungi^47^. Furthermore, the acetone crude extract of the stem bark of *Sclerocarya birrea* is a promising source for *anti-H pylori* compounds, with terpinen-4-ol, an essential oxygenated monoterpene oil, being the most abundant agent (35.83%), and it was reported as a major mediator of the *anti-H pylori* activity^48^. The inhibitory activity of terpinen-4-ol in this study was similar to that of amoxicillin, one of the most effective drugs used in the eradication of *H. pylori* infections worldwide^49^. Additionally, trans*-*sabinene hydrate, terpinen-4-ol, α-terpineol, and γ-terpinene have been reported as major components of marjoram oil, which exhibited antibacterial activity against food-related bacteria like *E. coli, Salmonella cholraesius*, and *S. aureus* in fresh sausage. Because of their antimicrobial activity against food-borne bacteria EOs could be added to food products to extend their shelf life, but changes in the taste, as well as formulation problems, could represent a problem there in^21^.

Linalool (8.5%), α-terpineol (4.4%), and linalyl acetate (4.2%) are considered the most important components of Myrtle oil that showed significant antimicrobial activities against *Salmonella typhimorium*, *Lactobacillus* spp., *Yersinia enterocolitica, Helicobacter pylori*^50^, and significant antifungal activity when combined with amphotericin B^51^. Moreover, the antimicrobial activity of the essential oil of *Thymus capitatus* was tested using the broth dilution method. γ-Terpinene in *Thymus capitatus* essential oil (10%) induced strong bactericidal activity against *H. pylori* strains^52^.

*β*-Caryophyllene, is a natural bicyclic sesquiterpene, which is extracted from clove and tested for the eradication of *H. pylori* in a mouse model, and its effects on the inflammation of the gastric mucosa. Interestingly, β-caryophyllene showed potent antimicrobial activity against *H. pylori* by direct killing action. In addition, it improved the inflammation of the gastric mucosa by decreasing *H. pylori* number^53^. The use of compounds with natural origin has gained popularity in scientific research focussed on drug innovation against *H. pylori* because of their broad flexibility and low toxicity. For example, monoterpenes limonene and *β*-pinene resulted in MICs against *H. pylori* of 75 µg/mL and 500 µg/mL respectively^54^.

Regarding the biological properties, we know that essential oils are complex mixtures of numerous constituents. As a result, their biological effects can be the result of the synergism of all their constituents, thus components are working together. In this case, the effect of the mixture would be greater than the pure sum of its single parts^55^. The essential oils could be used on their own, as well as in combination with other oils or synthetic active agents since synergy was observed by combining these substances. Various studies showed that the extent of antimicrobial activity and the mode of action is dependent on the additive, synergistic, or even antagonistic effects of the individual constituents.

Results of this study clearly showed the synergistic effect of both marjoram and petitgrain mandarin oils on their *anti-H. pylori* activity. The combined oil sample showed the highest inhibitory effect against *H. pylori* at MIC 1.95 µg/mL. Clarithromycin, the used reference drug, demonstrated the same MIC value as the combined oil, at the same concentration used. Thus, it should be noted that the combined oils' effects are comparable to clarithromycin.

An *in-silico* study was carried out to further verify the observed results. Docking studies are performed to reveal the binding affinity of the major components to the target enzymes^56^^,^^57^, where caryophyllene oxide showed the best fitting scores followed by linalyl acetate and methyl-*N*-methyl anthranilate. Furthermore, all the tested compounds showed high margins of safety *in-silico*, they were predicted to have no potential toxicity and were aligned with all Lipinski's rules. Additionally, all the tested compounds showed no or minimal interaction with microsomal cytochromes, thus, they could be taken concomitantly with other medications. Moreover, neither of the tested compounds was found to be a substrate for the p-glycoprotein (one of the resistance mechanisms of *H. pylori* for existing antibiotics)^40^ or had any reported Pan Assay Interference (PAINS). From this perspective, the two oil extracts are considered promising inhibitors for both sensitive and resistant strains of *H. pylori* with a notable safety margin and good desirable pharmacokinetic properties.

## 5. Conclusion

Marjoram and mandarin oils are the most widely available and highly consumed by humans due to their nutritional and medicinal values and very low toxic effects. The current study revealed the promising synergistic effects of the volatile constituents from marjoram and mandarin leaves against *Helicobacter pylori,* offering a natural remedy for pharmaceutical industries to combat this bacterial infection. Furthermore, the major compounds of both oils were evaluated *in-silico*, and demonstrated superior biological activities, excellent safety margins and desired pharmacokinetic properties making them excellent candidates to manage *H. pylori*.
